# Exploring the temporal development of childhood IgE profiles to allergen components

**DOI:** 10.1186/2045-7022-2-24

**Published:** 2012-12-19

**Authors:** Annica Önell, Lisbeth Hjälle, Magnus P Borres

**Affiliations:** 1 Phadia AB now Thermo Fisher Scientific, Uppsala, Sweden; 2 Department of Clinical and Experimental Medicine, Linköping University, Linköping, Sweden; 3 Astrid Lindgren Children’s Hospital, Karolinska University Hospital, Stockholm, Sweden; 4 Institute for Clinical Sciences, Sahlgrenska Academy of Göteborg University, Göteborg, Sweden

**Keywords:** Children, Atopic march, Specific IgE, ImmunoCAP ISAC, CRD, Allergen components

## Abstract

**Background:**

Children often develop allergies that may or not persist into adulthood. Although the different allergic symptoms over time have been well documented, the underlying pattern of sensitization to various proteins and subsequent allergy development is unexplored.

The aim was to study the sensitization pattern to allergen components over time from infancy to adulthood in a group of infants with heredity for allergic diseases.

**Methods:**

IgE profiles were monitored in a group of 67 children from 6 months to 18 years using a microarray chip (ImmunoCAP® ISAC) containing 103 allergen components derived from 47 allergen sources. The chip IgE profile was compared with clinical history, skin prick test results and diagnoses (atopic dermatitis, asthma and allergic rhinoconjunctivitis) at each time point for each child.

**Results:**

IgE profiles were unique for each child and showed broad agreement with the results of skin prick tests and doctors’ diagnoses. In addition, close examination of the IgE profiles often revealed early indication of subsequent allergies. IgE profiles also facilitated the examination of cross-reactivity contra co-sensitization, thereby greatly enhancing the possibility for managing patients.

**Conclusion:**

This explorative description indicates that sensitization pattern to allergen components differs over time as well as among allergic individuals when examined with microarray technology.

## Introduction

Atopic disease is one of the most common chronic disorders worldwide among both children and adults. The term “atopic march” refers to the dynamic process of manifestation, persistence, and remission of different atopic phenotypes in the first two decades of life [1,2]. Atopic dermatitis *per se* does not constitute a risk for asthma, but may do so when associated with allergic sensitization [3]. Persistent asthma is more likely in children with early sensitization to indoor allergens and exposure.

Objective measurement of atopic status, including quantification of markers of sensitization, is needed to quantify asthma risk [3,4]. Recent research has identified some useful indicators of potential allergy manifestation. These indicators include family history of asthma and allergies, early, multiple and severe sensitization to some food- and aeroallergens, and early viral infections [3]. Quantitative measures of atopy, especially cumulative titers of IgE to perennial inhalant allergens, provide more robust assessments of atopy-associated risk than simple binary classifications (such as sensitized or non-sensitized). Consequently, developing methods that identify early sensitization to allergens will provide a critical step in the management of allergy manifestation in children. Today, a substantial proportion of children who develop persistent asthma are not identified until their disease has already consolidated, thus preventing any potential benefits of early intervention.

Recent studies have shown that allergen microarray chips are novel tools for high-resolution IgE profiling in patients with atopic dermatitits [5,6], in adult multi-sensitized patients with respiratory symptoms [7], and leads to a more precise diagnosis of sensitization [8-10]. The chip technology enables a simultaneous determination of specific IgE against multiple allergens, using a minimal amount of serum [8,11]. Pediatric array studies have shown that cross-reacting pollen allergens are absent in the first years of life [12] and that multi-sensitization towards certain furry animal components are associated with increased bronchial inflammation in severe asthmatic children [13].

The aim of this study was to describe explorative the IgE profiles to allergen components in a group of infants over time.

## Methods

### Study population

Pregnant women (n = 67) in Linköping, Sweden were invited to participate [14]. The babies were classified into two groups: double heredity (n = 46) and no family history of atopic disease (n = 21). Double heredity was defined as atopic disease either in both parents or in one parent and one sibling.

A clinical examination was done, and a history was obtained regarding symptoms of allergies at 3, 6, 9, and 18 months of age, [14] at 6 years of age [15] and at 18 years of age [16]. The physical examination focused on symptoms of allergy. Venous blood was sampled, SPTs were performed and questionnaires were used at each occasion. In total, 64 children completed the study, out of which 49 were venipunctured at the last checkup. The study was approved by the regional ethics committee at Linköping University (# 03694).

### Physical examination

Atopic dermatitis was defined as proposed by Hanifin and Raijka [17] with the use of the modified criteria for young infants [18] and according to the SCORAD index [19]. For children under 18 years of age, asthma was defined as three or more episodes of bronchial obstruction verified by a physician [20]. At 18 years of age, asthma was defined according to the changes in the spirometric results in the exercise provocation [16].

Allergic rhinoconjunctivitis was defined as rhinitis or conjunctivitis appearing after exposure to a particular allergen and without any infection. The diagnosis was based on a history of relevant symptoms (itching in eyes or nose, tearing, eye redness, runny nose, sneezing and nasal obstruction) having occurred at least twice during the previous two years.

The clinical diagnosis of atopic dermatitis, asthma, and allergic rhinoconjunctivitis was made when children were 1.5, 6, and 18 years of age. The classification was based on physical examination and questionnaires. SPTs were performed with the following allergens; egg, milk, fish animal epithelia, birch, timothy grass, mugwort, house dust mite, Alternaria and Cladosporium described in detail elsewhere [14-16].

### Circulating IgE antibodies

Sera were analyzed using the ImmunoCAP ISAC 103 microarray chip, (Thermo Fisher Scientific, Vienna). It contains 103 allergen components derived from 47 allergen sources and used by allergy specialists and immunologists as a complement to other established diagnostic tests [21,22]. The testing procedure was carried out according to the manufacturer's instructions for use. The IgE results were reported in ISAC Standardized Units (ISU). The ISAC measurement range is 0.3 – 100 ISU. For each individual a descriptive analysis of data was carried out where each ISAC IgE response was compared with the patient’s clinical history, doctor’s diagnosis, and SPT at each time point.

## Results

**Figure 1:**
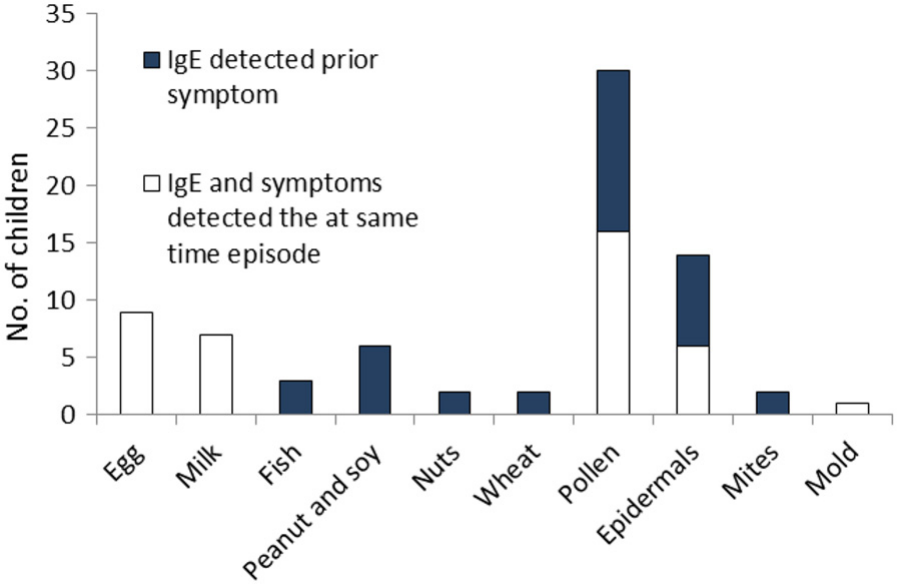
**Number of children sensitized to different allergens.** White bars represent children with IgE abs observed at the same time as symptoms were reported. Black bars represent children with IgE abs observed at least one time episode (see Methods) prior to any symptoms to the given allergen was reported.

Overall the clinical history and the ISAC IgE responses were in broad agreement. Of 82 triggering allergens causing symptoms as defined by doctors diagnosis, 76 (93%) were identified by the ISAC chip. An overview of the sensitization profile in the study group is presented in Figure 1. For each individual, sensitization to a given allergen was compared with any clinical reaction at the same or at a later time episode. For this purpose, a sensitization to a given allergen was only counted once per individual. Among the younger children, 6 to 18 months old, the top 3 most prevalent sensitizing allergen was hen’s egg (14%), followed by cow’s milk (11%), and peanut (6%). The most common egg components were Gal d 1 and 2 (n = 9). The most common milk allergen was Bos d 8, which usually occurred with concomitant sensitization to several other milk components (n = 7). The 4 subjects with IgE antibodies (hereafter, abs) to peanut components all revealed concomitant sensitization to Ara h 1, 2, and 3. Among the older children, 6 or 18 years old, the top 3 most prevalent sensitizing allergens were timothy (42%), birch (28%), and cat (22%). IgE abs to Phl p 1 to 5 were observed in 27 out of 64 children. Bet v 1 was the most prevalent component for birch (n = 18), and it always occurred with concomitant sensitization to several other PR-10 allergens from nuts and fruits. Bet v 1 was the most frequent PR-10 protein, followed by Cor a 1.0401, Mal d 1, Pru p 1, Ara h 8, Gly m 4, in descending order. The major cat allergen Fel d 1 was the most common cat component (n = 14). In young children, the most prevalent cross-reacting allergen group was serum albumin (n = 7, 14%). However, only three of the 1.5 year old children, and not always the same individuals as the seven 6 – 18 year old ones, displayed IgE to serum albumin. Conversely, PR-10 proteins were the most prevalent cross-reacting allergens among the older children (n = 18, 28%), while PR-10 sensitization was absent in 6 to 9 month old children. No detectable IgE abs was found to lipid transfer proteins or cross-reactive carbohydrate determinants (CCD). Profilin abs were detected only in 18-year-old subjects (n = 5). Tropomyosin abs were detected in two children at 1.5 year of age, one of them remained sensitized at 6 years of age, but none at 18 years age.

Overall the performance and quality of the ISAC results were acceptable for all patients and components except for rAsp f 1, nBos d 8 and nBos d lactoferrin, where assay backgrounds below < 1 ISU were registered for 20-25% of the measurements. Hence only responses ≥ 1 ISU were taken into consideration in the data analysis.

All children revealed unique IgE sensitization “fingerprints” over time. No child was similar to another even though they presented similar symptomatology. Four subgroups of patients with common features were identified based on the sensitisation pattern over time, see below. Eleven children were excluded from this subgroup classification due to missing time episode samples.

### Early multiple food and aeroallergen sensitized group (Early multi)

Ten children (19%) revealed IgE ab binding to egg and milk early in life (< 1.5 years), sometimes with parallel sensitization to fish or storage proteins from soy, peanut, or tree nuts. As these children aged (*i.e.*, became >1.5 years old), sensitization to aeroallergens such as pollen, animal dander, or mites, became prevalent, whereas milk and egg sensitization usually declined and vanished over time. All clinically diagnosed allergies in this group were confirmed by the chip results. All 10 children in this group revealed unique IgE profiles (e.g., see Additional file 1). By resolving co-sensitization for cross-reactivity and by identifying unexpected triggers prior to symptom development, the ISAC chip provided valuable information in 8 out of 10 children in the multi-sensitized group.

### Late sensitized group (Late IgE)

Twenty individuals (38%) did not reveal any sensitization to egg, milk, fish, soy, wheat or peanut early in life, but developed IgE abs to aeroallergens (pollen, mites, cat, or dog) later in life (at 6 years or older), with or without cross-reacting food allergens like PR-10 proteins. All diagnosed allergies were detected by the ISAC chip, except for 3 patients with SPT confirmed egg allergy. All 20 children in this group revealed unique IgE profiles (Additional file 2). Nine of 20 children showed relatively simple IgE profiles, with only one or two species-specific allergens (typically mono-sensitized to birch, grass, or cat). The remaining 11 children displayed a multi-sensitized IgE profile, involving cross-reacting allergens (often PR-10 proteins) with concomitant sensitization to at least 2 species-specific components. For these more complex, multi-sensitized children the ISAC results gave new, relevant information not easily available from SPT or case histories.

### Early food sensitized group (F)

Two children (4%) displayed a low-level response (< 2 ISU) to one of the egg components before 1.5 years of age, without developing any sensitization to aeroallergens later in life. The diagnosed egg allergy was confirmed by the chip for both children. In this case, the ISAC results did not add new, relevant information relative to traditional diagnostic methods. This group of patient was not studied further due to the limited number of individuals.

### Non-sensitized group (No IgE)

Twenty-one children (40%) did not reveal any IgE ab response. In this group of patients the ISAC chip missed two children with diagnosed egg allergy and confirming egg SPT, and one child with diagnosed cat and dog allergy and confirming SPT. Of these 21 children, only eight were diagnosed at least once with atopic dermatitis, asthma, or rhinoconjunctivitis during the 18 year follow-up. The ISAC results did not add new, relevant information relative to traditional diagnostic methods, except for delivering a rapid and reliable answer that the child is non-sensitized to a broad spectrum of allergens.

### Sensitization pattern versus clinical diagnosis

**Figure 2:**
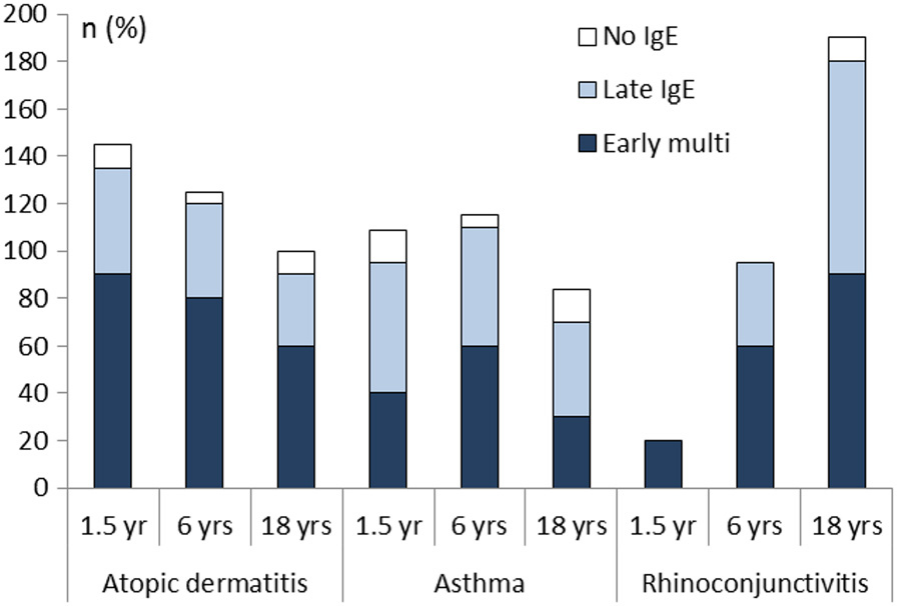
**Percentage of children in the three study groups (Early multiple food and aeroallergen sensitization, Late IgE and No IgE) diagnosed with atopic dermatitis, asthma, and allergic rhino-conjunctivitis at each time point.** (Since a child may have up to 3 defined symptoms *n* can end up to a maximum of 300%).

Children in the early multiple food and aeroallergen sensitized (Early Multi) group were more likely to have atopic dermatitis compared to those who later were sensitized (Late A) and non-sensitized at all ages (Figure 2). Early multi-sensitized children also developed allergic rhinoconjunctivitis at an earlier age compared to those who were only aeroallergen sensitized. The percentage of children who developed asthma did not differ between these two groups. Half of the children who were only sensitized to an aeroallergen also had non-IgE mediated atopic dermatitis. Children who were not sensitized were unlikely to have atopic dermatitis, asthma, or allergic rhinoconjunctivitis (range 0-14%).

The worst possible case scenario was for a subject to accumulate three diagnoses at each of the three time intervals, adding up to a maximum of nine diagnoses. The early multi-sensitized group accumulated a mean number of 5.3 diagnoses, the group with late sensitization a mean of 3.8 diagnoses, and the non-IgE group a mean of 0.7 diagnoses.

### Molecular spreading over time

**Figure 3:**
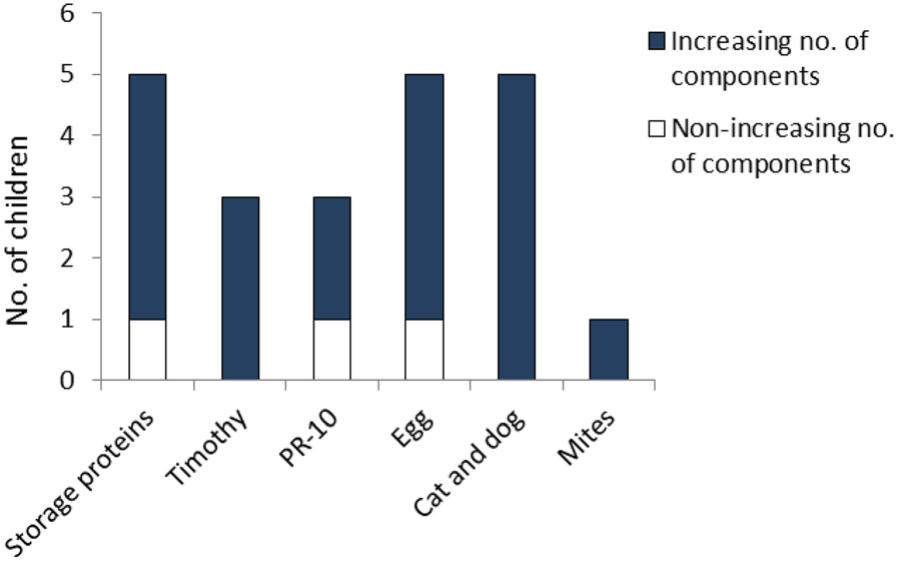
Number of children with increasing (blue, n = 19) and non-increasing (red, n = 3) IgE eliciting components in the initial phase of the IgE development process.

A number of offending allergens were identified with IgE ab responses prior to any reported clinical reactions to that allergen (Figure 1). We registered 22 observations with detectable IgE abs, with at least two consecutive time episodes, following the actual time episode to a given allergen source (Figure 3). In 19 of these 22 observations, an initial phase with an increasing number of sensitizing components was observed, typically with increasing IgE levels.

## Discussion

We describe in this explorative study that sensitization to allergen components in allergic children increase over time. Based on these findings, we hypothesize that sensitization profiling provides additional information regarding early identification, cross-reactivity, co-sensitization and unexpected triggers in allergic children.

The ISAC results in this study confirm that egg and milk were the most common sensitizing allergens among young children, while aeroallergen components from birch, grass, and cat were the most prevalent allergens among older children. Interestingly, serum albumin (SA) was the most common cross-reacting allergen among young children in our study. SA sensitization was always accompanied by IgE abs to several milk components, indicating that the SA sensitization in young children originates from the bovine SA, Bos d 6, present in milk. Melioli et. al. [12] reported PR-10 proteins to be the most prevalent cross-reacting protein among ten to eighteen years-old Italian children. Our results confirm that PR-10 proteins are the most prevalent cross-reacting allergens also in children from Sweden. In our population cross-reacting proteins like profilin and LTPs were absent in contrast to children from the Mediterranean area.

Two children with egg allergy and one with pet allergy were not detected with the ISAC chip. One reason could be that the test results are semiquantative compared to ImmunoCAP technology. If the recent ISAC chip with 112 components had been used, these children might have been detected as this chip has improved sensitivity.

The ISAC results showed good agreement with SPT and subsequent doctor’s diagnoses for each patient. In addition to confirming known allergies, the ISAC chip added previously unknown but relevant information in 8 of 10 children with multiple food and aeroallergen sensitization, and in 9 out of 20 children multi-sensitized to aeroallergens. The added information usually concerned resolving cross-reactivity from co-sensitization. This information was not easily extracted from data based on SPT and/or clinical history. These results are in agreement with previous studies that have shown the ISAC chip is especially useful in resolving IgE profiles in multi-sensitized patients, and also adds additional patient management value in about 30% of patients visiting allergy specialist clinics [23-25].

However, sensitization is not always accompanied with relevant allergic symptoms. Therefore only a thorough medical evaluation by a clinician can diagnose an allergy.

Interestingly, all individuals displayed unique IgE sensitization “fingerprints”. The individual fingerprints were also unique over time but we identified four subgroups with common IgE profile trends**.** Simpson et al have described that IgE antibody responses do not reflect a single phenotype of atopy, but a rather multiple different atopic vulnerabilities [26]. Only one of these atopic classes (multiple early atopic vulnerability) predicts asthma. We could not confirm this with our equivalent FA group, maybe due to limited sample size. However, we found that our equivalent group to multiple early atopic vulnerability developed allergic rhinoconjunctivitis early in life.

One interesting observation in this study was that we could document an increasing number of sensitizing components, often with concurrently increasing IgE levels in 19 of 22 evaluated children. Interestingly, the IgE abs were typically detected long before any clinical reactions were reported.

A major drawback with this study was the long sampling intervals, precluded monitoring the IgE production development phase in detail. A study design using a higher sampling frequency is needed in order to explore the precise developmental stages of allergies relative to IgE profiles. The strength of this study is the long follow-up period where allergy development up to young adulthood could be followed in all of the included infants.

Our results demonstrate that the allergen micro-array chip is a promising tool in allergy diagnostics, especially for multi-sensitized children with severe asthma and eczema. Based on our findings of this explorative study, we hypothesize that each child with an IgE mediated allergic disease develops its own unique allergic component “fingerprint” over time. With the component results available at the time of the medical examinations in the study, we speculate that the patient management would have been different for some of the children in this cohort. Further, a better understanding of the underlying causes of the symptoms would have been possible for many of the more complex, high risk children.

## Abbreviations

IgE: Immunoglobulin E; kUA/L: Kilounits of allergen-specific IgE per liter; SPT: Skin prick test; Abs: Antibodies; ISAC: Immunosolid-phase allergen chip; ISU: ISAC standardized units; FEV1: Forced expiratory volume in one second; CCD: Cross-reactive carbohydrate; CRD: Component Resolved Diagnosis; ARC: Allergic rhinoconjunctivitis; MA: Molecular allergology; SA: Serum albumins.

## Competing interests

Annica Önell and Magnus Borres are employed by Thermo Fisher Scientific, Uppsala, Sweden. Lisbeth Hjälle declares that she has no competing interests.

## Authors’ contributions

MB carried out the majority of the examinations except the 18 year follow up and drafted the manuscript. LH carried out the majority of cohort laboratory work and AÖ carried out ImmunoCAP ISAC analyses, the data analysis and drafted the manuscript. All authors read and approved the final manuscript.

## Authors’ information

MP Borres, MD, has been responsible for this cohort from the beginning to the 18 year follow up and carried out the majority of the examinations. L Hjalle has been participating in the planning, collection of the data during the entire period and carried out the majority of laboratory work. A Onell has been responsible for the ImmunoCAP ISAC analyses and the data analysis. All three has written the manuscript together.

## Supplementary Material

Additional file 1 Representative patient case with early multiple food and aeroallergens. Click here for file

Additional file 2 Representative patient case with late sensitization. Click here for file
