# Supplementary material for: Exploring the temporal development of childhood IgE profiles to allergen components
Source: Clin Transl Allergy. 2012 Dec 19;2:24. doi: 10.1186/2045-7022-2-24 (PMC3574828; doi:10.1186/2045-7022-2-24)
Supplement: Additional file 1 — Representative patient case with early multiple food and aeroallergens. [file 2045-7022-2-24-S1.docx]

**Table 1** Representative patient case with early multiple food and aeroallergens

| **Age:** | | | **6 months** | | | **9 months** | | | **18 months** | | | **6 years** | | |
| --- | --- | --- | --- | --- | --- | --- | --- | --- | --- | --- | --- | --- | --- | --- |
| **Symptoms:** | | | **Eczema and multiple viral infections** | | | | | | | | | **Asthma** | | |
|  | **Source** | **Component** | **ISAC** | **SPT** | **Symp** | **ISAC** | **SPT** | **Symp** | **ISAC** | **SPT** | **Symp** | **ISAC** | **SPT** | **Symp** |
| Food | Hazel nut | nCor a 9 | 0 |  |  | 0 |  |  | 0.6 |  |  | 0 |  |  |
|  | Sesame | nSes i 1 | 0 |  |  | 0 |  |  | 2.1 |  |  | 3.2 |  |  |
|  | Peanut | nAra h 1 | 0 |  |  | 0.3 |  |  | 0.8 |  |  | 6.3 |  | **Yes** |
|  |  | nAra h 2 | 3.8 |  |  | 5.7 |  |  | 7.5 |  |  | 6.4 |  |  |
|  |  | nAra h 3 | 0.4 |  |  | 1.3 |  |  | 1.1 |  |  | 0.9 |  |  |
|  | Egg | nGal d 1 | 1.1 | **Pos** | **Yes** | 3.9 | **Pos** | **Avoid** | 3.9 | **Pos** | **Avoid** | 0.4 | **Pos** | **No** |
|  |  | nGal d 2 | 0.6 |  |  | 1.4 |  |  | 2.5 |  |  | 0.5 |  |  |
|  | Milk | nBos d 8 | 0.4 | **Pos** | **Yes** | 0.4 | **Pos** | **Avoid** | 0.3 | **Pos** | **Avoid** | 0 | **Neg** | **No** |
|  |  | nBos d lactoferr | 0 |  |  | 0.5 |  |  | 0.3 |  |  | 0 |  |  |
|  | Carp | rCyp c 1 | 0 | **Neg** | **No** | 0 | **Neg** | **No** | 1.0 | **Pos** | **Avoid** | 0 | **Pos** | **Avoid** |
|  | Cod | rGad c 1 | 0 |  |  | 1.0 |  |  | 4.9 |  |  | 0 |  |  |
| Tropomyosin | Shrimp | rPen a 1 | 0 |  |  | 0 |  |  | 0 |  |  | 1.8 |  | **Yes** |
|  |  | nPen i 1 | 0 |  |  | 0 |  |  | 0 |  |  | 5.6 |  |  |
|  |  | nPen m 1 | 0 |  |  | 0 |  |  | 0 |  |  | 6.9 |  |  |
|  | Mite | rDer p 10 | 0 |  |  | 0 |  |  | 0 |  |  | 3.2 |  |  |
|  | Cockroach | nBla g 7 | 0 |  |  | 0 |  |  | 0 |  |  | 8.4 |  |  |
|  | Anisakis | rAni s 3 | 0 |  |  | 0 |  |  | 0 |  |  | 3.1 |  |  |
| Grass | Timothy | rPhl p 1 | 0 |  |  | 0 |  |  | 0 | **Neg** | **No** | 0.3 | **Neg** |  |
|  |  | nPhl p 4 | 0 |  |  | 0 |  |  | 0 |  |  | 1.5 |  |  |
|  |  | rPhl p 5 | 0 |  |  | 0 |  |  | 0 |  |  | 0.3 |  |  |
| PR-10 | Birch | rBet v 1 | 0 |  |  | 0 |  |  | 0 | **Neg** | **No** | 29.7 | **Pos** | **Yes** |
|  | Alder | rAln g 1 | 0 |  |  | 0 |  |  | 0 |  |  | 1.2 |  |  |
|  | Hazelpollen | rCor a 1.0101 | 0 |  |  | 0 |  |  | 0 |  |  | 1.2 |  |  |
|  | Hazel nut | rCor a 1.0401 | 0 |  |  | 0 |  |  | 0 |  |  | 6.0 |  |  |
|  | Apple | rMal d 1 | 0 |  |  | 0 |  |  | 0 |  |  | 3.8 |  |  |
|  | Peach | rPru p 1 | 0 |  |  | 0 |  |  | 0 |  |  | 0.7 |  |  |

**Legend to Table 1.**

Representative ISAC results for a male child with atopic eczema, food allergy, and asthma. Egg and milk allergy diagnoses were set at 6, 9, and 18 months with confirming SPT and ISAC results. An egg/milk free diet was followed until 6 years of age when he developed tolerance. Fish was introduced at 12 months and was diagnosed with fish allergy at 18 months. Birch pollen allergy was first diagnosed at 6 years, with confirming SPT and reported OAS-like symptoms to peanut and shellfish (although these reactions were never confirmed with any diagnostic test before ISAC). ISAC results are reported in ISU, 0.3-1 ISU (yellow), 1-15 (orange and >15 (red). SPT results are reported as positive (Pos) or negative (Neg). When avoiding a causing food allergen this is marked under symptoms (Avoid).
