# Supplementary material for: Exploring the temporal development of childhood IgE profiles to allergen components
Source: Clin Transl Allergy. 2012 Dec 19;2:24. doi: 10.1186/2045-7022-2-24 (PMC3574828; doi:10.1186/2045-7022-2-24)
Supplement: Additional file 2 — Representative patient case with late sensitization. [file 2045-7022-2-24-S2.docx]

**Table 2** Representative patient case with late sensitization

|  |  | **Age:** | **6 months** | | | **18 months** | | | **6 years** | | | **18 years** | | |
| --- | --- | --- | --- | --- | --- | --- | --- | --- | --- | --- | --- | --- | --- | --- |
|  | **Symptoms:** | | **Healthy** | | | **Wheeze** | | | **Asthma, RC** | | | **Rhinoconjunct.** | | |
|  | **Source** | **Component** | **ISAC** | **SPT** | **Symp** | **ISAC** | **SPT** | **Symp** | **ISAC** | **SPT** | **Symp** | **ISAC** | **SPT** | **Symp** |
| Grass pollen | Bermuda | nCyn d 1 | 0 |  |  | 0 | Neg |  | 0 | Neg |  | 33.2 | **Pos** | **Yes** |
|  | Timothy | rPhl p 1 | 0 |  |  | 0 |  |  | 0.6 |  |  | 34.8 |  |  |
|  |  | nPhl p 4 | 0 |  |  | 0 |  |  | 5.0 |  |  | 26.7 |  |  |
|  |  | rPhl p 5 | 0 |  |  | 0 |  |  | 1.4 |  |  | 50.1 |  |  |
|  |  | rPhl p 6 | 0 |  |  | 0 |  |  | 0 |  |  | 11.6 |  |  |
|  |  | rPhl p 11 | 0 |  |  | 0 |  |  | 0 |  |  | 12.8 |  |  |
| PR-10 | Birch | rBet v 1 | 0 |  |  | 0 | Neg |  | 0 | Neg |  | 46.8 | **Pos** | **Yes** |
|  | Alder | rAln g 1 | 0 |  |  | 0 |  |  | 0 |  |  | 7.2 |  |  |
|  | Hazel pollen | rCor a 1.01 | 0 |  |  | 0 |  |  | 0 |  |  | 2.8 |  |  |
|  | Hazel nut | rCor a 1.04 | 0 |  |  | 0 |  |  | 0 |  |  | 10.7 |  |  |
|  | Apple | rMal d 1 | 0 |  |  | 0 |  |  | 0 |  |  | 3.1 |  |  |
|  | Peach | rPru p 1 | 0 |  |  | 0 |  |  | 0 |  |  | 2.9 |  |  |
|  | Peanut | rAra h 8 | 0 |  |  | 0 |  |  | 0 |  |  | 1.0 |  |  |
|  | Cat | rFel d 1 | 0 |  |  | 0 | Neg |  | 0 | Neg |  | 2.4 | **Pos** | **Yes** |
| Mite | House dust mite | nDer f 1 | 0 |  |  | 0 | Neg |  | 0.5 | Neg |  | 12.5 | **Pos** | **Yes** |
|  |  | nDer p 1 | 0 |  |  | 0 |  |  | 0.8 |  |  | 3.5 |  |  |
|  |  | nDer f 2 | 0 |  |  | 0 |  |  | 0.5 |  |  | 18.3 |  |  |
|  |  | nDer p 2 | 0 |  |  | 0 |  |  | 0.4 |  |  | 16.3 |  |  |
|  | Storage mite | rEur m 2 | 0 |  |  | 0 |  |  | 0 |  |  | 3.5 |  |  |

**Legend to Table 2.**

Representative ISAC result for a healthy boy at 6 months who later in life developed airway symptoms. At 18 years of age he tested positive and was diagnosed with allergy to grass, birch, cat, dog and mite. ISAC results are reported in ISU, 0.3-1 ISU (yellow), 1-15 (orange and >15 (red). SPT results are reported as positive (Pos) or negative (Neg).
